# Risk Factors, Treatment and Prognosis of Patients with Lung Cancer after Heart Transplantation

**DOI:** 10.3390/life11121344

**Published:** 2021-12-04

**Authors:** Karsten M. Heil, Matthias Helmschrott, Fabrice F. Darche, Tom Bruckner, Philipp Ehlermann, Michael M. Kreusser, Andreas O. Doesch, Wiebke Sommer, Gregor Warnecke, Norbert Frey, Rasmus Rivinius

**Affiliations:** 1 Department of Cardiology, Angiology and Pneumology, Heidelberg University Hospital, 69120 Heidelberg, Germany; karsten.m.heil@gmail.com (K.M.H.); matthias.helmschrott@med.uni-heidelberg.de (M.H.); fabrice.darche@med.uni-heidelberg.de (F.F.D.); philipp.ehlermann@med.uni-heidelberg.de (P.E.); michael.kreusser@med.uni-heidelberg.de (M.M.K.); andreas.doesch@gmail.com (A.O.D.); norbert.frey@med.uni-heidelberg.de (N.F.); 2 German Center for Cardiovascular Research (DZHK), Partner Site Heidelberg/Mannheim, 69120 Heidelberg, Germany; 3 Institute for Medical Biometry, University of Heidelberg, 69120 Heidelberg, Germany; bruckner@imbi.uni-heidelberg.de; 4 Department of Pneumology and Oncology, Asklepios Hospital, 36433 Bad Salzungen, Germany; 5 Department of Cardiac Surgery, Heidelberg University Hospital, 69120 Heidelberg, Germany; wiebke.sommer@med.uni-heidelberg.de (W.S.); gregor.warnecke@med.uni-heidelberg.de (G.W.)

**Keywords:** heart transplantation, immunosuppression, malignancy, mortality, lung cancer, survival

## Abstract

Long-term survival after heart transplantation (HTX) is impacted by adverse effects of immunosuppressive pharmacotherapy, and post-transplant lung cancer is a common occurrence. This study aimed to examine the risk factors, treatment, and prognosis of patients with post-transplant lung cancer. We included 625 adult patients who received HTX at Heidelberg Heart Center between 1989 and 2018. Patients were stratified by diagnosis and staging of lung cancer after HTX. Analysis comprised donor and recipient characteristics, medications including immunosuppressive drugs, and survival after diagnosis of lung cancer. A total of 41 patients (6.6%) were diagnosed with lung cancer after HTX, 13 patients received curative care and 28 patients had palliative care. Mean time from HTX until diagnosis of lung cancer was 8.6 ± 4.0 years and 1.8 ± 2.7 years from diagnosis of lung cancer until last follow-up. Twenty-four patients (58.5%) were switched to an mTOR-inhibitor after diagnosis of lung cancer. Multivariate analysis showed recipient age (HR: 1.05; CI: 1.01–1.10; *p* = 0.02), COPD (HR: 3.72; CI: 1.88–7.37; *p* < 0.01), and history of smoking (HR: 20.39; CI: 2.73–152.13; *p* < 0.01) as risk factors for post-transplant lung cancer. Patients in stages I and II had a significantly better 1-year (100.0% versus 3.6%), 2-year (69.2% versus 0.0%), and 5-year survival (53.8% versus 0.0%) than patients in stages III and IV (*p* < 0.01). Given the poor prognosis of late-stage post-transplant lung cancer, routine reassessment of current smoking status, providing smoking cessation support, and intensified lung cancer screening in high-risk HTX recipients are advisable.

## 1. Introduction

Heart transplantation (HTX) remains the standard of care for patients with irreversible end-stage heart failure [1,2]. Over the last decades, post-transplant survival has continuously been improving due to advances in surgical management, clinical experience, and immunosuppressive drug therapy [3,4,5,6]. The focus of post-transplant care has consequently been shifting from early risks such as acute rejection or surgical complications (bleeding, thromboembolic events, and wound infections) towards late sequelae such as cardiac allograft vasculopathy or neoplasms [7,8,9].

While great effort has been put into the diagnosis and management of cardiac allograft vasculopathy [9,10], the area of malignancies after HTX remains largely unexplored, especially given the vast spectrum of tumor entities [11,12,13,14,15,16]. Cutaneous malignancies and post-transplant lymphoproliferative disorders are frequently observed post-transplant tumors [17,18,19,20,21,22,23,24], but lung cancer is the most common solid organ malignancy after HTX [25,26,27,28,29].

Several risk factors for lung cancer after HTX have previously been discussed, including higher recipient age, male sex, tobacco smoking, chronic obstructive pulmonary disease, and coronary artery disease [18,19,20,21,22,23,24,30]. In addition to these general risk factors, HTX recipients require immunosuppressive pharmacotherapy to prevent rejection, which is usually higher-dosed than in other solid organ transplant recipients and known to increase the risk for neoplasms [11,12,13,14,15,16,17,18,19,20,21,22,23,24,25,26,27,28,29,30].

As a consequence of the elevated cardiovascular risk profile, many HTX recipients are at high risk for post-transplant lung cancer. However, post-transplant lung cancer is still poorly characterized among HTX recipients. The relative importance of risk factors, the role of the underlying immunosuppressive drug therapy, and the clinical course of patients with post-transplant lung cancer are barely understood. We therefore sought to investigate the risk factors, treatment, and prognosis of patients with lung cancer after HTX. Special emphasis was put on the clinical presentation of patients with lung cancer after HTX including location, classification, histology, and staging of lung cancer after HTX as well as the possible influence of post-transplant drug regimens, including immunosuppressive agents.

## 2. Patients and Methods

### 2.1. Patients

This study was performed in accordance with the ethical standards of the Declaration of Helsinki. Approval was granted by the institutional review board (IRB) of Heidelberg University (ethics approval number: S-286/2015, Version 1.2, 28 July 2020). We obtained written informed consent from patients for their inclusion in the Heidelberg HTX Registry and the clinical and scientific use of their data. The ethics approval does not require additional consent for this observational study as only routine clinical data were used [30,31,32,33,34,35,36,37].

All adult patients (≥18 years) who received HTX at Heidelberg Heart Center, Heidelberg, Germany, between 1989 and 2018 were included in this study, except for patients who had undergone HTX more than once. Minimum follow-up data of two years after HTX was obtained from all patients (data available until 1 January 2021). We initially stratified patients by diagnosis of lung cancer after HTX: patients with diagnosed lung cancer after HTX (“lung cancer group”) and patients without diagnosed lung cancer after HTX (“no lung cancer group”). Patients in the lung cancer group were further subdivided by stage (I-IV) according to the 8th edition of the Union for International Cancer Control (UICC 8) [38].

### 2.2. Follow-Up

Patient follow-up was performed according to Heidelberg Heart Center’s routine clinical protocol. After the initial hospital stay following surgery, patients were seen as outpatients in the HTX clinic monthly during the first six months after HTX, then bimonthly until the end of the first year, and approximately three to four times per year thereafter (with additional visits when clinically required) [30,31,32,33,34,35,36,37].

During follow-up, patients were asked about their current medication history, the presence of B symptoms (fever, night sweats, and weight loss), smoking behavior, and other signs and symptoms of cancer. In addition, routine follow-up included medical history, physical examination, 12-lead electrocardiogram (ECG), echocardiography, endomyocardial biopsy, annual chest X-ray, and blood tests including immunosuppressive drug monitoring [30,31,32,33,34,35,36,37].

### 2.3. Post-Transplant Medication

Post-transplant medication including immunosuppressive drug therapy was administered as per the center’s usual standard of care. Perioperatively, patients received an anti-thymocyte globulin-based immunosuppression induction therapy. Cyclosporine A and azathioprine were used as the initial immunosuppressive regimen prior to 2001. From 2001, mycophenolate mofetil replaced azathioprine subsequently and tacrolimus replaced cyclosporine A from 2006 onward. Steroids were tapered incrementally during the initial post-transplant months and were discontinued six months after HTX (unless clinically needed) [30,31,32,33,34,35,36,37].

### 2.4. Statistical Analysis

Data were analyzed using SAS (Version 9.4, SAS Institute, Cary, NC, USA) and expressed as mean ± standard deviation (SD) or as count (n) with percentage (%). For measures of association, difference of mean or hazard ratio (HR) with 95% confidence interval (CI) were used. Depending on the variable type and question, we used Student’s *t*-test, Mann–Whitney U-test, analysis of variance (ANOVA), Kruskal–Wallis test, chi-squared test, or Fisher’s exact test, as appropriate. The Kaplan–Meier estimator was utilized to graphically display 5-year post-transplant survival. A *p*-value of <0.05 was considered statistically significant [30,31,32,33,34,35,36,37].

We used univariate analyses to search for intergroup differences including recipient data, previous open-heart surgery, principal diagnosis for HTX, donor data, transplant sex mismatch, perioperative data, post-transplant drug regimen, and immunosuppressive drug therapy. Patients with lung cancer after HTX were further analyzed with regard to signs and symptoms of lung cancer after HTX. Characterization also included lung cancer location, classification, histology, staging, and treatment modalities. Our analysis of risk factors for lung cancer after HTX included a multivariate analysis (Cox regression model) with the following five clinically relevant parameters based on a predetermined model: recipient age (years), recipient coronary artery disease (in total), recipient peripheral artery disease (in total), recipient chronic obstructive pulmonary disease (in total), and recipient history of smoking (in total). We did not include additional parameters in this multivariate analysis of risk factors for lung cancer after HTX in order to avoid biased regression coefficients and to ensure a stable number of events (patients with diagnosis of post-transplant lung cancer) per analyzed variable [30,31,32,33,34,35,36,37].

The primary outcome of this study was mortality after diagnosis of post-transplant lung cancer in patients after HTX. Secondary outcomes included analysis of risks factors and treatment of post-transplant lung cancer in patients after HTX.

## 3. Results

### 3.1. Baseline Characteristics and Medication after Heart Transplantation

Of 625 included HTX recipients, 41 patients (6.6%) were diagnosed with post-transplant lung cancer while 584 patients (93.4%) were not diagnosed with lung cancer during the study period. The mean interval from HTX to initial diagnosis of post-transplant lung cancer was 8.6 ± 4.0 years and 1.8 ± 2.7 years from diagnosis of lung cancer until last follow-up.

Patients with lung cancer after HTX had a significantly higher recipient age (55.4 ± 8.4 years versus [vs.] 51.7 ± 10.5 years, difference: 3.7 years, 95% confidence interval [CI]: 0.9–6.5 years, *p* = 0.01), a significantly higher percentage of coronary artery disease (24 of 41 [58.5%] vs. 232 of 584 [39.7%]; difference: 18.8%, 95% CI: 3.2–34.4%; *p* = 0.02), a significantly higher percentage of peripheral artery disease (13 of 41 [31.7%] vs. 71 of 584 [12.2%]; difference: 19.5%, 95% CI: 5.0–34.0%; *p* < 0.01), a significantly higher percentage of chronic obstructive pulmonary disease (16 of 41 [39.0%] vs. 135 of 584 [23.1%]; difference: 15.9%, 95% CI: 0.6–31.2%; *p* = 0.02), a significantly higher percentage of history of smoking (40 of 41 [97.6%] vs. 341 of 584 [58.4%]; difference: 39.2%, 95% CI: 33.0–45.4%; *p* < 0.01), and a significantly higher number of pack years (20.2 ± 10.5 py vs. 12.0 ± 14.4 py, difference: 8.2 py, 95% CI: 4.7–11.7 py, *p* < 0.01). We found no statistically significant differences between both groups with regard to the remaining recipient data, previous open-heart surgery, principal diagnosis for HTX, donor data, transplant sex mismatch, or perioperative data (all *p* ≥ 0.05). Baseline characteristics of study participants are summarized in Table 1.

Analysis of the immunosuppressive drug regimen showed no statistically significant difference between patients with and without lung cancer after HTX with regard to the use of cyclosporine A, tacrolimus, azathioprine, or mycophenolate mofetil (all *p* ≥ 0.05). As azathioprine is known to be the immunosuppressant with the highest impact on cancer, we compared lung cancer patients with or without azathioprine with regard to the percentage of lower (stages I and II) and higher stages (stages III and IV) of post-transplant lung cancer. There was no statistically significant difference between lung cancer patients with or without azathioprine regarding lower (azathioprine: 7 of 23 [30.4%] versus no azathioprine: 6 of 18 [33.3%]; *p* = 0.843) or higher stages of lung cancer after HTX (azathioprine: 16 of 23 [69.6%] versus no azathioprine: 12 of 18 [66.7%]; *p* = 0.843). We also did not observe statistically significant differences between patients with and without lung cancer after HTX correlating with the initial blood concentration of cyclosporine A or tacrolimus as the target drug trough blood levels of calcineurin inhibitors changed over time and were higher in the past.

In terms of concomitant medications, we observed no statistically significant differences between patients with and without lung cancer after HTX with respect to the administration of acetylsalicylic acid, beta-blockers, ivabradine, calcium channel blockers, angiotensin-converting-enzyme inhibitors/angiotensin II receptor blockers, or statins (all *p* ≥ 0.05). An overview of medications after HTX is given in Table 2.

### 3.2. Clinical Presentation of Patients with Lung Cancer after Heart Transplantation

In 26 of 41 patients (63.4%) with lung cancer after HTX, the diagnosis was made after the occurrence of at least one of the following symptoms: shortness of breath (26 of 41 [63.4%]), fatigue (22 of 41 [53.7%]), cough (22 of 41 [53.7%]), rust-colored sputum (19 of 41 [46.3%]), loss of weight (19 of 41 [46.3%]), night sweats (16 of 41 [39.0%]), infection (15 of 41 [36.6%]), fever (14 of 41 [34.1%]), loss of appetite (14 of 41 [34.1%]), and hoarseness (6 of 41 [14.6%]). Lung cancer after HTX was incidentally detected in 15 of 41 patients (36.6%), either on chest X-ray (12 of 41 [29.3%]) or on chest computed tomography (3 of 41 [7.3%]). Of note, none of the patients with post-transplant lung cancer had a known life-time history of malignancy prior to HTX.

Regarding smoking status, 40 of 41 patients (97.6%) diagnosed with lung cancer after HTX had a history of smoking before HTX. In order to be listed for HTX, patients had to quit smoking and had to be tobacco product free for a minimum of six months. After HTX, 35 of 41 patients (85.4%) who developed post-transplant lung cancer had started smoking again. Clinical presentation of patients with lung cancer after HTX is shown in Table 3.

### 3.3. Location, Classification, Histology, and Staging of Lung Cancer after Heart Transplantation

Lung cancer after HTX was predominantly located in the left upper lobe (17 of 41 [41.5%]), followed by the right upper lobe (11 of 41 [26.8%]), and the right lower lobe (9 of 41 [22.0%]), whereas localization in the left lower lobe (3 of 41 [7.3%]) or in the right middle lobe (1 of 41 [2.4%]) was less common. The most common classification of lung cancer after HTX was non-small-cell lung cancer (38 of 41 [92.7%]) with only a minority of small-cell lung cancer (3 of 41 [7.3%]). The main histological types were squamous-cell carcinoma (20 of 41 [48.8%]) and adenocarcinoma (16 of 41 [39.0%]), while neuroendocrine carcinoma (3 of 41 [7.3%]) and large-cell carcinoma (2 of 41 [4.9%]) were rarely found.

According to UICC 8 staging, the majority of patients were diagnosed at stage IV (19 of 41 [46.3%]) and at stage III (9 of 41 [22.0%]), whereas only a minority of patients were diagnosed at stage I (8 of 41 [19.5%]) and at stage II (5 of 41 [12.2%]). Location, classification, histology, and staging of lung cancer after HTX are provided in Table 4.

### 3.4. Prognosis and Treatment of Patients with Lung Cancer after Heart Transplantation

About one-third of patients diagnosed with lung cancer after HTX (13 of 41 [31.7%]) received treatment with a curative aim, while the other two-thirds (28 of 41 [68.3%]) received palliative care. Treatment mainly consisted of palliative radiotherapy (25 of 41 [61.0%]) and palliative chemotherapy (20 of 41 [48.8%]). Surgical treatment was performed in 16 of 41 patients with lung cancer after HTX (39.0%), whereas adjuvant chemotherapy and adjuvant radiotherapy (each 5 of 41 [12.2%]) as well as neoadjuvant chemotherapy and neoadjuvant radiotherapy (each 3 of 41 [7.3%]) were less common.

A switch to a mechanistic target of rapamycin [mTOR]-inhibitor was performed in over half of patients after diagnosis of post-transplant lung cancer (24 of 41 [58.5%]). The majority of these patients received everolimus (20 of 24 [83.3%]). As everolimus has only been available in Germany since 2004, sirolimus was used off-label prior to this in selected patients (4 of 24 [16.7%]). In addition, four patients (4 of 41 [9.8%]) were already on everolimus before diagnosis of post-transplant lung cancer as they showed loss of kidney function under calcineurin inhibitors. Prognosis and treatment of patients with lung cancer after HTX is presented in Table 5.

### 3.5. Multivariate Analysis of Risk Factors for Lung Cancer after Heart Transplantation

In order to assess the impact of different variables on lung cancer after HTX, we performed a multivariate analysis of risk factors for lung cancer after HTX. Out of five analyzed variables, the following three were significantly associated with the occurrence of lung cancer after HTX: higher recipient age (HR: 1.05, CI: 1.01–1.10; *p* = 0.02), chronic obstructive pulmonary disease (HR: 3.72, CI: 1.88–7.37; *p* < 0.01), and history of smoking (HR: 20.39, CI: 2.73–152.13; *p* < 0.01). Coronary artery disease (HR: 1.26, CI: 0.64–2.49; *p* = 0.50) and peripheral artery disease (HR: 0.75, CI: 0.36–1.57; *p* = 0.45) were not statistically significant. Multivariate analysis of risk factors for lung cancer after heart HTX is shown in Table 6.

### 3.6. Survival after Post-Transplant Lunger Cancer

**Figure 1:**
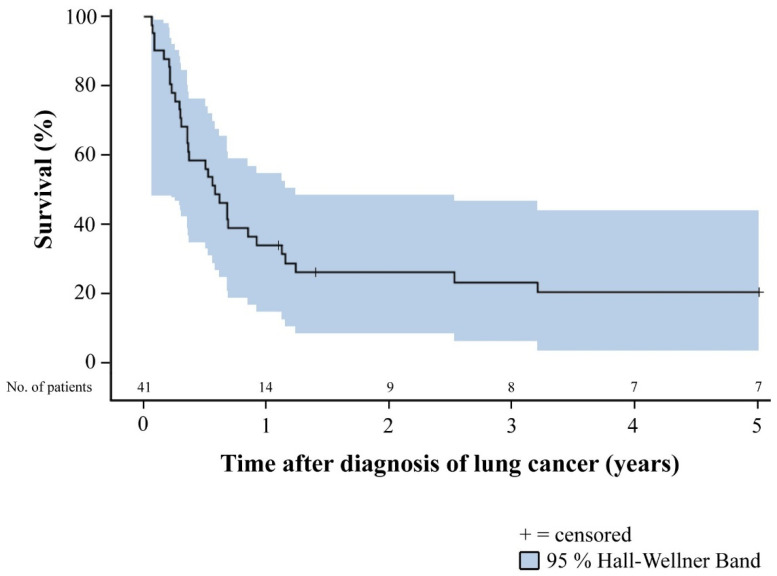
Overall 5-year survival of patients with diagnosis of lung cancer after HTX (Kaplan–Meier estimator). Patients with diagnosis of lung cancer after HTX had in general a poor prognosis. Overall 1-year (34.1%), 2-year (22.0%), and 5-year survival (17.1%) of patients with diagnosis of lung cancer after HTX were markedly reduced. Abbreviations: HTX = heart transplantation.

**Figure 2:**
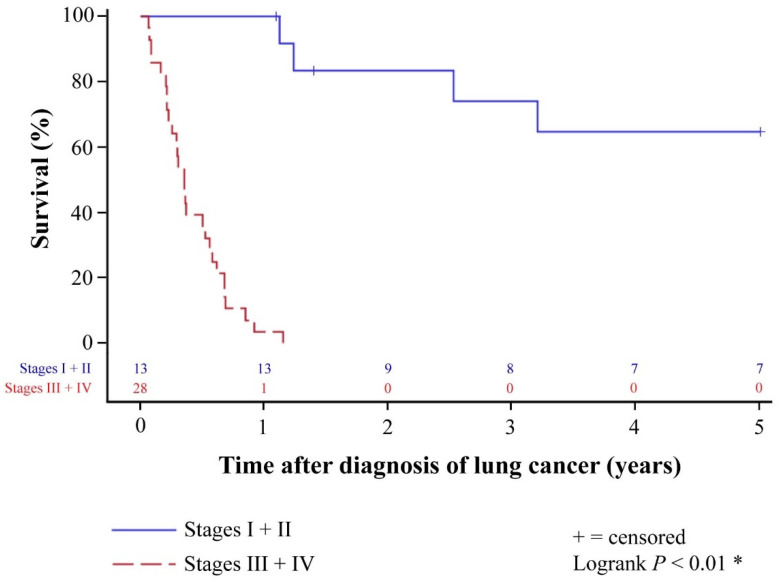
The 5-year survival of patients with diagnosis of lung cancer after HTX stratified by stage according to UICC 8 (Kaplan–Meier estimator). Patients in stage I and II lung cancer after HTX had a significantly better 1-year (100.0% versus 3.6%), 2-year (69.2% versus 0.0%), and 5-year survival (53.8% versus 0.0%) than patients in stage III and IV lung cancer after HTX (*p* < 0.01). Abbreviations: HTX = heart transplantation; UICC 8 = 8th edition of the Union for International Cancer Control. * = statistically significant (*p* < 0.05).

Patients with lung cancer after HTX generally had a poor prognosis. Overall 1-year (14 of 41 [34.1%]), 2-year (9 of 41 [22.0%]), and 5-year survival (7 of 41 [17.1%]) of patients with diagnosis of lung cancer after HTX were markedly reduced. All patients who were deceased within five years after diagnosis of post-transplant lung cancer died from lung cancer or associated pulmonary infection. None of these patients died from cardiac allograft vasculopathy, acute rejection, or another cancer entity. Stratified by stage according to UICC 8, patients in stage I and II lung cancer after HTX had a significantly better 1-year (100.0% versus 3.6%), 2-year (69.2% versus 0.0%), and 5-year survival (53.8% versus 0.0%) than patients in stage III and IV lung cancer after HTX (*p* < 0.01). Overall 5-year survival of patients with diagnosis of lung cancer after HTX as well as 5-year survival of patients with diagnosis of lung cancer after HTX stratified by stage according to UICC 8 is displayed in Figure 1 and Figure 2.

## 4. Discussion

### 4.1. Frequency and Significance of Lung Cancer after Heart Transplantation

Post-transplant lung cancer is the most common solid organ malignancy after HTX and plays an important role in the long-term care of HTX recipients [25,26,27,28,29]. Nevertheless, the field lacks sufficient data on the underlying risk factors, the available treatment options, and the clinical prognosis of patients with lung cancer after HTX. Given this need, we performed this large study with a total of 625 HTX recipients to characterize patients with post-transplant lung cancer in detail. Forty-one patients (6.6%) were diagnosed with post-transplant lung cancer and mean time from HTX until diagnosis of lung cancer was 8.6 ± 4.0 years.

Previous studies published post-transplant lung cancer rates ranging from 1.6% to 5.8% [18,19,20,21,22,23,24,26,28,39]. These numbers clearly highlight the increased risk for the development of lung cancer in patients after HTX compared to other solid-organ transplant recipients and the general population [18,19,23,26,28]. Earlier studies have differed in length of post-transplant follow-up and consequently in time between HTX and the diagnosis of cancer, which may have caused an underestimation of post-transplant lung cancer rates. Pham and colleagues [23] reported a post-transplant lung cancer rate of 1.6% (10 of 608) with a mean interval from HTX to lung cancer detection of 2.3 ± 1.5 years, whereas in our study, we observed a higher post-transplant lung cancer rate of 6.6% (41 of 625) with a longer mean interval from HTX to lung cancer detection of 8.6 ± 4.0 years.

Furthermore, in some studies, post-transplant lung cancer was already diagnosed within the first year after HTX [18,20,21,22]. Given the rather slow growth rate of lung cancer, these tumors were likely to preexist before HTX [20]. In contrast, no patient was diagnosed with post-transplant lung cancer within 24 months after HTX in our study, which might be the result of extensive pre-transplant screening for lung cancer including the presence of B symptoms, smoking behavior, medical history, physical examination, and chest X-ray/computed tomography. Considering the above-mentioned frequencies and intervals to diagnosis, post-transplant lung cancer is a long-term HTX sequela of considerable importance.

### 4.2. Risk Factors for Lung Cancer after Heart Transplantation

As HTX recipients generally require a higher-dosed immunosuppressive drug regimen than other solid organ transplant recipients to prevent rejection, they seem to have an elevated risk to develop neoplasms [18,19,20,21,22,23,24,25,26,27,28,29]. Calcineurin inhibitors (cyclosporine A or tacrolimus)—which are commonly used as standard immunosuppressants in HTX recipients—in particular are associated with an increased rate of neoplasms and can also promote tumor progression via direct cellular effects [20,40,41,42]. However, we did not observe a statistically significant difference regarding the use of cyclosporine A or tacrolimus between HTX recipients with or without post-transplant lung cancer. This may indicate that immunosuppressive drug therapy itself does not necessarily cause a higher occurrence of post-transplant lung cancer but rather enhances preexisting risk factors [18,22]. HTX recipients are a selected group of patients with an elevated risk profile including higher age, male sex, history of smoking, chronic obstructive pulmonary disease, and ischemic cardiomyopathy [18,19,20,21,22,23,24,30].

In our study, we found that higher recipient age (HR: 1.05; CI: 1.01–1.10; *p* = 0.02), chronic obstructive pulmonary disease (HR: 3.72; CI: 1.88–7.37; *p* < 0.01), and history of smoking (HR: 20.39; CI: 2.73–152.13; *p* < 0.01) are significant risk factors for post-transplant lung cancer. Patients with lung cancer after HTX were also more likely to be of male sex (87.8% versus 77.7%) and have ischemic cardiomyopathy (43.9% versus 32.5%), but this difference was not statistically significant (*p* = 0.13 respectively *p* = 0.14).

Of the discussed factors, smoking appears to be by far the most important risk factor for lung cancer after HTX [18,19,20,21,22,23,24]. Furthermore, smoking may potentiate the adverse effects of the underlying immunosuppressive drug therapy, especially in patients who restarted smoking after HTX. In our study, 40 of 41 patients with lung cancer after HTX smoked before HTX (97.6%) and 35 of these 41 patients started smoking again after HTX (85.4%). We therefore strongly recommend asking patients about their smoking status before and after HTX as well as providing assistance and guidance to those patients who started smoking again in order to ensure a permanent cessation of smoking.

### 4.3. Clinical Outcomes of Patients with Lung Cancer after Heart Transplantation

The overall survival of HTX recipients with post-transplant lung cancer is mostly quite poor [18,19,21,23,24,27,28]. We observed an overall 1-year survival rate of HTX recipients of 34.1% (14 of 41), an overall 2-year survival rate of 22.0% (9 of 41), and an overall 5-year survival rate of 17.1% (7 of 41) after diagnosis of post-transplant lung cancer. This is in line with findings by Pham and colleagues [23] who found an overall 2-year survival rate of 22.0%, and by Crespo-Leiro and colleagues [19] who reported an overall 5-year survival rate of 16.0%. A possible explanation for these low survival rates could be the fact that many HTX recipients are diagnosed at an advanced stage of disease [18,19,20,21,22,23,24].

When stratified by stage according to UICC 8, patients in stages I and II lung cancer after HTX showed a significantly better 1-year (100.0% versus 3.6%), 2-year (69.2% versus 0.0%), and 5-year survival (53.8% versus 0.0%) than patients in stages III and IV in our study. Similar findings were published by Crespo-Leiro and colleagues [19] who found a superior 2-year survival (70.0% versus 16.0%) in patients with curative surgery, by Bruschi and colleagues [18] who reported a significantly better 5-year survival (56.0% versus 0.0%) in patients with early-stage lung cancer after HTX, and by Bagan and colleagues [27] who showed a significantly better 5-year survival (44.0% versus 0.0%) in patients with N0 stage.

These data indicate that a 5-year survival after diagnosis of post-transplant lung cancer of about 50% can be achieved in HTX recipients if the following three criteria are fulfilled [18,19,20,23,27]: First, lung cancer after HTX is detected at an early stage (preferably stages I and II). Second, curative treatment (especially surgery) is feasible as surgical resection is the treatment of choice for lung cancer. Third, prompt and aggressive treatment of lung cancer is initiated in order to prevent lymph node involvement and metastases [18,19,20,23,27].

Whereas criteria two and three strongly depend on the patient’s health status and underlying comorbidities, the first criterion may be improved by intensified lung cancer screening as patients are often asymptomatic at the time of lung cancer detection [18,19,20,21,22]. Standard lung cancer screening in HTX recipients mainly consists of annual chest X-rays, however, the sensitivity of this method to detect early-stage lung cancer is rather low [19,22,27]. In addition, if an abnormality is found on chest X-ray, it is often misinterpreted as an infectious process rather than a cancerous process [21]. Low-dose chest computed tomography shows promising results as an additional screening modality for the early detection of post-transplant lung cancer [18,19,20,21,22,27]. Therefore, high-risk HTX recipients (higher age, male sex, history of smoking, chronic obstructive pulmonary disease, and ischemic cardiomyopathy) may benefit from an intensified lung cancer screening including low-dose chest computed tomography.

### 4.4. Study Limitations

The results of this study were based on a single-center registry (Heidelberg HTX Registry) including the highly detailed data of 625 HTX recipients who received HTX at Heidelberg Heart Center. As this study design has certain limitations, our findings should be interpreted with caution and within the context of the existing literature. Nevertheless, we would like to emphasize that our study was comparable to multicenter studies in sample size and our patients received standardized treatment and follow-up, lowering the likelihood of potential confounders and selection bias [14,30,31,32,33,34,35,36,37].

Considering the slow growth of these tumors, long-term follow-up is essential to detect lung cancer after HTX. We therefore decided to include adult HTX recipients who received HTX at Heidelberg Heart Center between 1989 and 2018, ensuring a minimum follow-up of two years after HTX. Given the long study period, a possible era effect as a result of changes in medical and surgical care may have affected our findings. As tacrolimus replaced cyclosporine A as the main immunosuppressive agent from 2006 onward, the maximum possible follow-up time of patients with tacrolimus was shorter than of patients with cyclosporine A. However, both groups had a mean follow-up time of more than five years after HTX, supporting the robustness of our results [14,30,31,32,33,34,35,36,37].

Our findings should be seen as hypothesis-generating, particularly in the context of risk factors for post-transplant lung cancer and mortality after HTX as multiple factors can influence these outcomes. Large multi-center trials are required to confirm our findings.

## 5. Conclusions

Many HTX recipients have a distinct cardiovascular risk profile with an elevated risk for post-transplant lung cancer. Specifically, higher recipient age, chronic obstructive pulmonary disease, and history of smoking are significant risk factors for lung cancer after HTX. Notably, 35 of 41 patients (85.4%) who developed post-transplant lung cancer in this study had started smoking again after HTX. Given the particular risk for patients who take up smoking again after HTX, it is advisable to intensify efforts to ensure continued abstinence. This should begin with reassessment of smoking status at every follow-up encounter after HTX as well as offering guidance and assistance with smoking cessation if needed.

We observed an enormous difference in survival between patients diagnosed in an early stage of lung cancer after HTX and those diagnosed later. Patients with early-stage lung cancer after HTX showed a 5-year survival rate of more than fifty percent after diagnosis of post-transplant lung cancer, which is comparable to non-transplant patients with early-stage lung cancer [43], while patients with late-stage lung cancer after HTX had a very poor prognosis. The dramatically differing survival strongly suggests that early diagnosis is key. Thorough screening for lung cancer should start prior to HTX and continue regularly afterward. In addition to annual chest X-rays, low-dose chest computed tomography in high-risk HTX recipients may be a viable option to intensify screening efforts. However, comprehensive data on this are currently lacking and further research is needed before changing clinical practice.

## Figures and Tables

**Table 1:** Baseline characteristics.

Parameter	All (n = 625)	No Lung Cancer after HTX (n = 584)	Lung Cancer after HTX (n = 41)	Difference	95% CI	*p*-Value	
**Recipient data**							
Age (years), mean ± SD	52.0 ± 10.4	51.7 ± 10.5	55.4 ± 8.4	3.7	0.9–6.5	0.01	*
Male sex, n (%)	490 (78.4%)	454 (77.7%)	36 (87.8%)	10.1%	−0.5–20.7%	0.13	
Body mass index (kg/m^2^), mean ± SD	24.9 ± 4.0	24.9 ± 4.0	24.9 ± 3.3	0.0	−1.1–1.1	0.97	
Arterial hypertension, n (%)	342 (54.7%)	314 (53.8%)	28 (68.3%)	14.5%	−0.3–29.3%	0.07	
Dyslipidemia, n (%)	397 (63.5%)	366 (62.7%)	31 (75.6%)	12.9%	−0.8–26.6%	0.10	
Diabetes mellitus, n (%)	211 (33.8%)	194 (33.2%)	17 (41.5%)	8.3%	−7.3–23.9%	0.28	
Coronary artery disease ^†^, n (%)	256 (41.0%)	232 (39.7%)	24 (58.5%)	18.8%	3.2–34.4%	0.02	*
Peripheral artery disease, n (%)	84 (13.4%)	71 (12.2%)	13 (31.7%)	19.5%	5.0–34.0%	<0.01	*
COPD, n (%)	151 (24.2%)	135 (23.1%)	16 (39.0%)	15.9%	0.6–31.2%	0.02	*
History of smoking, n (%)	381 (61.0%)	341 (58.4%)	40 (97.6%)	39.2%	33.0–45.4%	<0.01	*
Pack years (py), mean ± SD	12.6 ± 14.3	12.0 ± 14.4	20.2 ± 10.5	8.2	4.7–11.7	<0.01	*
Renal insufficiency ^, n (%)	358 (57.3%)	333 (57.0%)	25 (61.0%)	4.0%	−11.5–19.5%	0.62	
eGFR (mL/min/1.73 m^2^), mean ± SD	60.4 ± 21.7	60.5 ± 22.0	59.5 ± 16.1	1.0	−4.3–6.3	0.72	
**Previous open-heart surgery**							
Overall open-heart surgery, n (%)	183 (29.3%)	175 (30.0%)	8 (19.5%)	10.5%	−2.2–23.2%	0.16	
CABG surgery, n (%)	78 (12.5%)	73 (12.5%)	5 (12.2%)	0.3%	−10.1–10.7%	0.95	
Other surgery °, n (%)	70 (11.2%)	67 (11.5%)	3 (7.3%)	4.2%	−4.2–12.6%	0.41	
VAD surgery, n (%)	48 (7.7%)	48 (8.2%)	0 (0.0%)	8.2%	−0.6–10.8%	0.06	
**Principal diagnosis for HTX**							
Ischemic CMP, n (%)	208 (33.3%)	190 (32.5%)	18 (43.9%)	11.4%	−4.3–27.1%	0.14	
Non-ischemic CMP, n (%)	383 (61.3%)	361 (61.8%)	22 (53.7%)	8.1%	−7.6–23.8%	0.30	
Valvular heart disease, n (%)	34 (5.4%)	33 (5.7%)	1 (2.4%)	3.3%	−1.8–8.4%	0.38	
**Donor data**							
Age (years), mean ± SD	41.0 ± 13.5	41.3 ± 13.5	37.4 ± 13.0	3.9	−0.4–8.2	0.07	
Male sex, n (%)	271 (43.4%)	249 (42.6%)	22 (53.7%)	11.1%	−4.7–26.9%	0.17	
Body mass index (kg/m^2^), mean ± SD	24.8 ± 4.1	24.8 ± 4.1	24.2 ± 3.6	0.6	−0.6–1.8	0.27	
**Transplant sex mismatch**							
Mismatch, n (%)	280 (44.8%)	266 (45.5%)	14 (34.1%)	11.4%	−3.7–26.5%	0.16	
Donor (m) to recipient (f), n (%)	30 (4.8%)	30 (5.1%)	0 (0.0%)	5.1%	−3.6–7.2%	0.14	
Donor (f) to recipient (m), n (%)	250 (40.0%)	236 (40.4%)	14 (34.1%)	6.3%	−8.7–21.3%	0.43	
**Perioperative data**							
Ischemic time (min), mean ± SD	222.4 ± 68.6	223.3 ± 69.0	209.4 ± 61.8	13.9	−6.3–34.1	0.17	
Biatrial HTX, n (%)	164 (26.2%)	152 (26.0%)	12 (29.3%)	3.3%	−11.1–17.7%	0.65	
Bicaval HTX, n (%)	184 (29.5%)	173 (29.6%)	11 (26.8%)	2.8%	−11.3–16.9%	0.70	
Total orthotopic HTX, n (%)	277 (44.3%)	259 (44.4%)	18 (43.9%)	0.5%	−15.2–16.2%	0.96	

Abbreviations: CABG = coronary artery bypass graft; CI = confidence interval; CMP = cardiomyopathy; COPD = chronic obstructive pulmonary disease; f = female; eGFR = estimated glomerular filtration rate; HTX = heart transplantation; m = male; n = number; py = pack year; SD = standard deviation; VAD = ventricular assist device; ^†^ = presence of coronary artery disease before HTX; ^ = eGFR < 60 mL/min/1.73 m^2^; ° = congenital, valvular or ventricular surgery; * = statistically significant (*p* < 0.05).

**Table 2:** Initial medications after HTX.

Parameter	All (n = 625)	No Lung Cancer after HTX (n = 584)	Lung Cancer after HTX (n = 41)	Difference	95% CI	*p*-Value
**Immunosuppressive drug therapy**						
Cyclosporine A, n (%)	347 (55.5%)	319 (54.6%)	28 (68.3%)	13.7%	−1.1–28.5%	0.09
Tacrolimus, n (%)	278 (44.5%)	265 (45.4%)	13 (31.7%)	13.7%	−1.1–28.5%	0.09
Azathioprine, n (%)	267 (42.7%)	244 (41.8%)	23 (56.1%)	14.3%	−1.4–30.0%	0.07
Mycophenolate mofetil, n (%)	358 (57.3%)	340 (58.2%)	18 (43.9%)	14.3%	−1.4–30.0%	0.07
Steroids, n (%)	625 (100.0%)	584 (100.0%)	41 (100.0%)	0.0%	n. a.	n. a.
**Concomitant medications**						
ASA, n (%)	64 (10.2%)	58 (9.9%)	6 (14.6%)	4.7%	−6.4–15.8%	0.34
Beta blocker, n (%)	113 (18.1%)	107 (18.3%)	6 (14.6%)	3.7%	−7.6–15.0%	0.55
Ivabradine, n (%)	54 (8.6%)	52 (8.9%)	2 (4.9%)	4.0%	−3.0–11.0%	0.38
Calcium channel blocker, n (%)	165 (26.4%)	150 (25.7%)	15 (36.6%)	10.9%	−4.3–26.1%	0.13
ACE inhibitor/ARB, n (%)	276 (44.2%)	253 (43.3%)	23 (56.1%)	12.8%	−2.9–28.5%	0.11
Diuretic, n (%)	625 (100.0%)	584 (100.0%)	41 (100.0%)	0.0%	n. a.	n. a.
Statin, n (%)	241 (38.6%)	222 (38.0%)	19 (46.3%)	8.3%	−7.5–24.1%	0.29
Gastric protection †, n (%)	625 (100.0%)	584 (100.0%)	41 (100.0%)	0.0%	n. a.	n. a.

Abbreviations: ASA = acetylsalicylic acid; ACE inhibitor = angiotensin-converting-enzyme inhibitor; ARB = angiotensin II receptor blocker; CI = confidence interval; HTX = heart transplantation; n = number; n. a. = not applicable; † = gastric protection defined as proton pump inhibitor (PPI) or histamine receptor (H2) blocker.

**Table 3:** Clinical presentation of patients with lung cancer after HTX.

Parameter	Lung Cancer after HTX (n = 41)
**Symptomatic**	
Symptomatic finding, n (%)	26 (63.4%)
Shortness of breath, n (%)	26 (63.4%)
Fatigue, n (%)	22 (53.7%)
Cough, n (%)	22 (53.7%)
Rust-colored sputum, n (%)	19 (46.3%)
Loss of weight, n (%)	19 (46.3%)
Night sweats, n (%)	16 (39.0%)
Infection, n (%)	15 (36.6%)
Fever, n (%)	14 (34.1%)
Loss of appetite, n (%)	14 (34.1%)
Hoarseness, n (%)	6 (14.6%)
**Asymptomatic**	
Asymptomatic finding, n (%)	15 (36.6%)
Incidental detection in chest X-ray, n (%)	12 (29.3%)
Incidental detection in chest computed tomography, n (%)	3 (7.3%)
**Smoking status**	
Smoking before HTX, n (%)	40 (97.6%)
Smoking after HTX, n (%)	35 (85.4%)

Abbreviations: HTX = heart transplantation; n = number.

**Table 4:** Location, classification, histology, and staging of lung cancer after HTX.

Parameter	Lung Cancer after HTX (*n* = 41)
**Location of lung cancer**	
Right upper lobe, n (%)	11 (26.8%)
Right middle lobe, n (%)	1 (2.4%)
Right lower lobe, n (%)	9 (22.0%)
Left upper lobe, n (%)	17 (41.5%)
Left lower lobe, n (%)	3 (7.3%)
**Classification of lung cancer**	
Small-cell lung cancer, n (%)	3 (7.3%)
Non-small-cell lung cancer, n (%)	38 (92.7%)
**Histology of lung cancer**	
Neuroendocrine carcinoma, n (%)	3 (7.3%)
Adenocarcinoma, n (%)	16 (39.0%)
Squamous-cell carcinoma, n (%)	20 (48.8%)
Large-cell carcinoma, n (%)	2 (4.9%)
**Tumor (T) Staging**	
T1a, n (%)	0 (0.0%)
T1b, n (%)	0 (0.0%)
T1c, n (%)	1 (2.4%)
T2a, n (%)	9 (22.0%)
T2b, n (%)	7 (17.1%)
T3, n (%)	8 (19.5%)
T4, n (%)	16 (39.0%)
**Lymph Node (N) Staging**	
N0, n (%)	9 (22.0%)
N1, n (%)	6 (14.6%)
N2, n (%)	14 (34.1%)
N3, n (%)	12 (29.3%)
**Metastasis (M) Staging**	
M0, n (%)	22 (53.7%)
M1a, n (%)	8 (19.5%)
M1b, n (%)	1 (2.4%)
M1c, n (%)	10 (24.4%)
**Stage according to UICC 8**	
IA, n (%)	1 (2.4%)
IB, n (%)	7 (17.1%)
IIA, n (%)	0 (0.0%)
IIB, n (%)	5 (12.2%)
IIIA, n (%)	3 (7.3%)
IIIB, n (%)	3 (7.3%)
IIIC, n (%)	3 (7.3%)
IVA, n (%)	10 (24.4%)
IVB, n (%)	9 (22.0%)

Abbreviations: HTX = heart transplantation; M = metastasis; N = lymph node; n = number; T = tumor; UICC 8 = 8th edition of the Union for International Cancer Control.

**Table 5:** Prognosis and treatment of patients with lung cancer after HTX.

Parameter	Lung Cancer after HTX (*n* = 41)
**Prognosis**	
Curative care, n (%)	13 (31.7%)
Palliative care, n (%)	28 (68.3%)
**Treatment**	
Neoadjuvant chemotherapy, n (%)	3 (7.3%)
Neoadjuvant radiotherapy, n (%)	3 (7.3%)
Surgery, n (%)	16 (39.0%)
Adjuvant chemotherapy, n (%)	5 (12.2%)
Adjuvant radiotherapy, n (%)	5 (12.2%)
Palliative chemotherapy, n (%)	20 (48.8%)
Palliative radiotherapy, n (%)	25 (61.0%)
**Change of immunosuppressive drug therapy**	
Switch from cyclosporin A to everolimus in combination with an antimetabolite, n (%)	12 (29.3%)
Switch from cyclosporin A to sirolimus in combination with an antimetabolite, n (%)	3 (7.3%)
Switch from tacrolimus to everolimus in combination with an antimetabolite, n (%)	7 (17.1%)
Switch from tacrolimus to sirolimus in combination with an antimetabolite, n (%)	0 (0.0%)
Switch to everolimus monotherapy with steroids, n (%)	1 (2.4%)
Switch to sirolimus monotherapy with steroids, n (%)	1 (2.4%)

Abbreviations: HTX = heart transplantation; n = number.

**Table 6:** Multivariate analysis of risk factors for lung cancer after HTX.

Variable	Hazard Ratio	95% CI	*p*-Value	
Recipient age (years)	1.05	1.01–1.10	0.02	*
Coronary artery disease (in total)	1.26	0.64–2.49	0.50	
Peripheral artery disease (in total)	0.75	0.36–1.57	0.45	
COPD (in total)	3.72	1.88–7.37	< 0.01	*
History of smoking (in total)	20.39	2.73–152.13	< 0.01	*

Abbreviations: CI = confidence interval; COPD = chronic obstructive pulmonary disease; HTX = heart transplantation; * = statistically significant (*p* < 0.05).

## Data Availability

Not applicable.
